# Transport of environmental natural organic matter coated silver nanoparticle across cell membrane based on membrane etching treatment and inhibitors

**DOI:** 10.1038/s41598-020-79901-y

**Published:** 2021-01-12

**Authors:** Laijin Zhong, Sisi Chen, Zhijie Tang, Xuewen Guo, Xin Hu, Weijuan Zheng, Hong-zhen Lian

**Affiliations:** 1 State Key Laboratory of Analytical Chemistry for Life Science, School of Chemistry & Chemical Engineering and Centre of Materials Analysis, Nanjing University, Nanjing, 210023 People’s Republic of China; 2 State Key Laboratory of Pharmaceutical Biotechnology, School of Life Science, Nanjing University, Nanjing, 210023 People’s Republic of China

**Keywords:** Environmental chemistry, Chemical safety, Environmental chemistry

## Abstract

Environmental natural organic matters (NOMs) have great effects on the physicochemical properties of engineering nanoparticles, which may impact the transport of nanoparticles across plasma membrane and the cytotoxicity. Therefore, the kinetics, uptake pathway and mass of transporting into A549 cell membrane of silver nanoparticles (AgNPs) coated with citric acid (CA), tartaric acid (TA) and fulvic acid (FA) were investigated, respectively. CA, FA and TA enhanced the colloidal stability of AgNPs in culture medium and have greatly changed the surface plasmon resonance spectrum of AgNPs due to the absorption of CA, FA and TA on surface of AgNPs. Internalizing model showed that velocity of CA-, TA- and FA-nAg transporting into A549 cell were 5.82-, 1.69- and 0.29-fold higher than those of the control group, respectively. Intracellular mass of Ag was dependent on mass of AgNPs delivered to cell from suspension, which obeyed Logistic model and was affected by NOMs that CA- and TA-nAg showed a large promotion on intracellular mass of Ag. The lipid raft/caveolae-mediated endocytosis (LME) of A549 cell uptake of AgNPs were susceptible to CA, TA and FA that uptake of CA-, TA- and FA-nAg showed lower degree of dependent on LME than that of the control (uncoated AgNPs). Actin-involved uptake pathway and macropinocytosis would have less contribution to uptake of FA-nAg. Overall, transmembrane transport of NOMs-coated AgNPs differs greatly from that of the pristine AgNPs.

## Introduction

The increasing application of engineering nanoparticles will inevitably result in the accumulation of these engineering nanoparticles in environment and may result in potential ecological and health risks^1–3^. For example, the accumulation of silver nanoparticles (AgNPs) can inhibit embryo growth^1^ and cause a series of cytotoxicity such as gene mutation^4^, inhibition of cell proliferation^5^, apoptosis^6,7^ and necrosis^8^. Now in vitro cytotoxicity investigations are frequently used to explore the toxic mechanism of nanoparticles^9–12^. The cytotoxicity of hardly soluble nanoparticles such as AgNPs mainly caused by intracellular particles according to “Trojan-horse mechanism”^5,13^. Therefore, many studies have been carried out on quantitative or qualitative analysis of intracellular nanoparticles to reveal cellular uptake of nanoparticles. Qualitative methods (e.g. Transmission electron microscopy^14^, Scanning electron microscopy^15^, Light scattering microscopy^16^, Super-resolution fluorescence microscopy^17^, Atomic force microscopy^18^) have been fully studied to directly observe intracellular nanoparticles. However, the quantitative methods of nanoparticles entering into cell are developed slowly compared to the qualitative methods^19^. The main challenge is how to erase the disruption of cell surface associated nanoparticles which are hard to be differentiated from intracellular nanoparticles^14,19–22^. Therefore, selective removal methods of cell surface associated nanoparticles with etchants have been developed^20,21^. The etchant I_2_-KI was firstly used to selectively remove gold nanoparticle (AuNPs) from cells and the internalized mass of Au nanoparticle was successfully analyzed^20^. The etchant K_3_Fe(CN)_6_-Na_2_S_2_O_3_ was proved to effectively remove silver nanoparticles from cell surface^21,23^. Therefore, mass of cellular nanoparticles can be quantitatively estimated via the removal of cell surface associated nanoparticles with etchants.

Typically uptake pathway for nanoparticles are macropinocytosis and endocytosis including clathrin mediated endocytosis (CME), lipid raft/caveolae-mediated endocytosis (LME)^24–28^. The nanoparticles transported through macropinocytosis or CME into cell will be usually transferred into lysosomal where the releasing ions from the insoluble particles are occurred^25,27,29^, so-called “lysosomal enhanced Trojan-horse mechanism”^5^. However, particles entering into cell through LME would sometimes escape degradation by lysosomal and release ions into cytoplasm or reach to organelle^24,26,28^. Inhibitors have been widely used to reveal the uptake pathway of nanoparticles^4,24,30^. Now, many researches have addressed the contribution of a certain uptake pathway to the nanoparticles via the decrease of particles’ signal with the addition of inhibitor^4,24,30^. However, these researches generally ignore interferences of the cell surface association nanoparticles which may lead to errors. Thus, we considered the combination etching method with inhibitors to analyse uptake pathway of nanoparticles.

The physicochemical properties of nanoparticles such as size, charge, and functionalization play a key role on their cellular uptake^8,25,31–35^. Before resuspending and inhaling by human beings, AgNPs entering into environment are inevitably contacted with nature organic matters (NOMs) such as citric acid (CA), tartaric acid (TA) and fulvic acid (FA)^36,37^. NOMs may be absorbed on the surface of AgNPs and change the surface properties, even the size and morphology of AgNPs as our previous report^37^. Cellular uptake and cytotoxicity of pristine nanoparticles have been well documented^1,8,19,24^, therefore, more investigations should be carried out on NOMs-nanoparticles corona to reveal the mechanism of the cellular uptake of AgNPs influenced by environmental NOMs.

Human pulmonary adenocarcinoma cell (A549 cell), a common model cell strain, was usually used to explore the cytotoxicity of nanoparticles^38,39^. In the present study, A549 cell were exposed to polyvinyl pyrrolidone (PVP) coated AgNPs (p-nAg) with size of around 20 nm with/without the treatment of CA, TA or FA. The p-nAg treated with solution without any NOM was set as control (nAg_control_). CA-, FA-, TA-coated nAg were marked as CA-nAg, FA-nAg and TA-nAg, respectively. A etchant (K_3_Fe(CN)_6_-Na_2_S_2_O_3_) was chosen to selectively remove AgNPs associated on cell surface of A549 and inhibitors of cytochalasin D (inhibiting actin involved uptake pathway), EIPA (inhibiting macropinocytosis), chlorpromazine (inhibiting CME) and filipin (inhibiting LME) were also used to investigate the uptake pathway. The aim of this study is to reveal the effects of NOMs on the cellular uptake of AgNPs.

## Results

### Characterization of NOMs-coated AgNPs and their stability in culture medium

**Figure 1:**
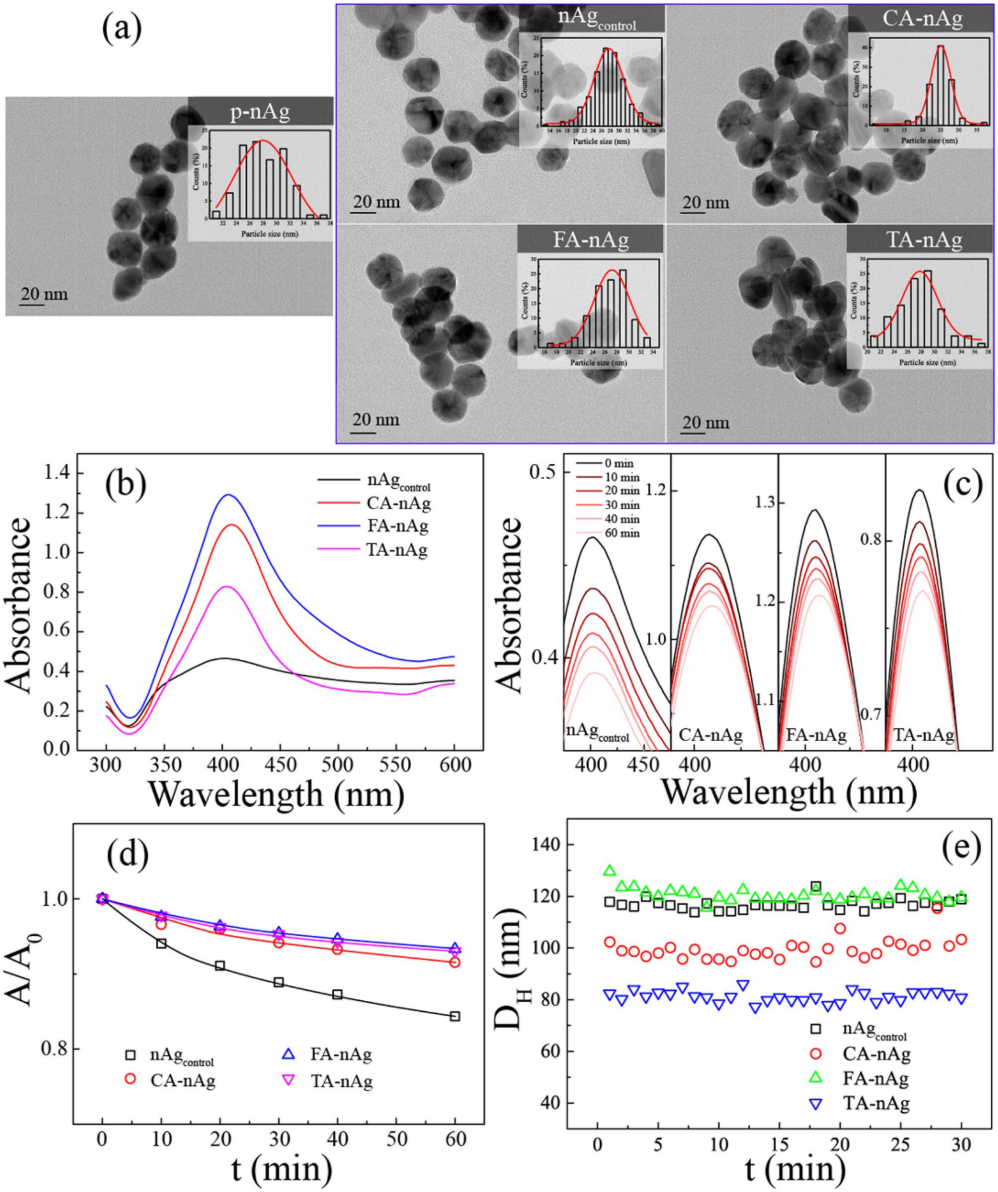
TEM characterizations of AgNPs and stability of AgNPs suspension in CM (20 μg ml^−1^ at 37 °C). (**a**) Size distribution of pristine nAg (p-nAg), nAg_control_, CA-nAg, FA-nAg and TA-nAg characterized by TEM at least 200 particles. (**b**) UV–Vis absorption spectrum of nAg_control_, CA-nAg, FA-nAg and TA-nAg suspension. (**c**) UV–Vis spectra over 60 min. (**d**) The ratios of the absorbance to the initial absorbance at λ_max_ over 60 min fitted with first order removal model. (**e**) Hydrodynamic diameters of AgNPs suspension over 30 min by DLS.

After treated with CA, FA or TA, the size of silver nanoparticle was little changed (25.1 nm to 27.7 nm in average) as shown in Fig. 1a. This was consistent with our previous study^37^. Table 1 showed that D_H_ in culture medium (CM) with 1% FBS of CA-nAg was substantially lower than D_H_ of nAg_control_ (99 nm to 117 nm in average). CA was absorbed on the surface of AgNPs and resulted in a higher carbon contents (1.91% to 1.77%) and higher ratio of content of carbon to content of nitrogen (C:N, wt:wt) compared to nAg_control_ (6.69 to 5.10) as shown in Table 1. Moreover, Table 1 showed that zeta potential value of CA-nAg in water (− 45.0 mV) was much negative than nAg_control_ in water (− 24.2 mV) since CA absorbed on the surface of AgNPs.

**Table 1:** Physicochemical properties of AgNPs.

	nAg_control_	CA-nAg	FA-nAg	TA-nAg
C (wt, %)	1.77	1.91	2.64	1.47
N (wt, %)	0.35	0.29	0.34	0.21
C:N (wt:wt)	5.10:1	6.69:1	7.80:1	7.12:1
D_T_^a^ (nm)	27.9 ± 7.9	25.1 ± 6.3	27.2 ± 7.0	27.8 ± 6.2
**D**_**H**_^**b**^** (nm)**				
W^d^	249 ± 10	54 ± 1	105 ± 1	68 ± 3
CM^e^	117 ± 2	99 ± 4*	121 ± 3	81 ± 2*
**ZP**^**c**^** (mV)**				
W	− 24.2 ± 10.8	− 45.0 ± 2.9*	− 42.7 ± 7.9*	− 28.8 ± 5.2
CM	− 0.8 ± 1.4	− 1.7 ± 0.9	− 0.9 ± 2.3	− 2.9 ± 3.7

^a^Diameter of AgNPs confirmed by TEM (count from 200 particles).

^b^Zeta-potential value.

^c^Hydrodynamic diameter of AgNPs in solution.

^d^Water.

^e^Culture medium supported with 1% FBS.

*Signed to the significant difference from control (p < 0.05).

Surface plasmon resonance (SPR) spectrum of NOMs-coated AgNPs suspensions in CM (20 μg ml^−1^) at 37 °C was shown in Fig. 1b. The maximum absorption wavelength (λ_max_) of nAg_control_, CA-, FA- and TA-nAg were 402, 408, 406 and 404 nm, respectively. Figure 1c,d recorded the SPR spectrums of suspensions within 60 min and the trends of absorption value at λ_max_ (A_max_) within 60 min. Red-shift of λ_max_ for all of suspensions depended on contact time and A_max_ trended to decrease. Within 60 min, λ_max_ of nAg_control_, CA-, FA- and TA-nAg was red-shift for 2 nm and their A_max_ decreased for 15.6, 8.4, 6.6 and 7.0%, respectively. The ratio of real time absorbance to initial absorbance (A/A_0_) at λ_max_ over 60 min could be fitted well with the first order removal model, which is a simplified model for particles sedimentation in water solution^40^. The model details and fitting parameters were listed in SI (Table S1). However, Fig. 1e showed that D_H_ of these AgNPs in CM were almost constant within 30 min. It implied that these AgNPs suspended stable in CM in dilute concentration.

### Localization of NOMs-coated AgNPs in A549 cell

**Figure 2:**
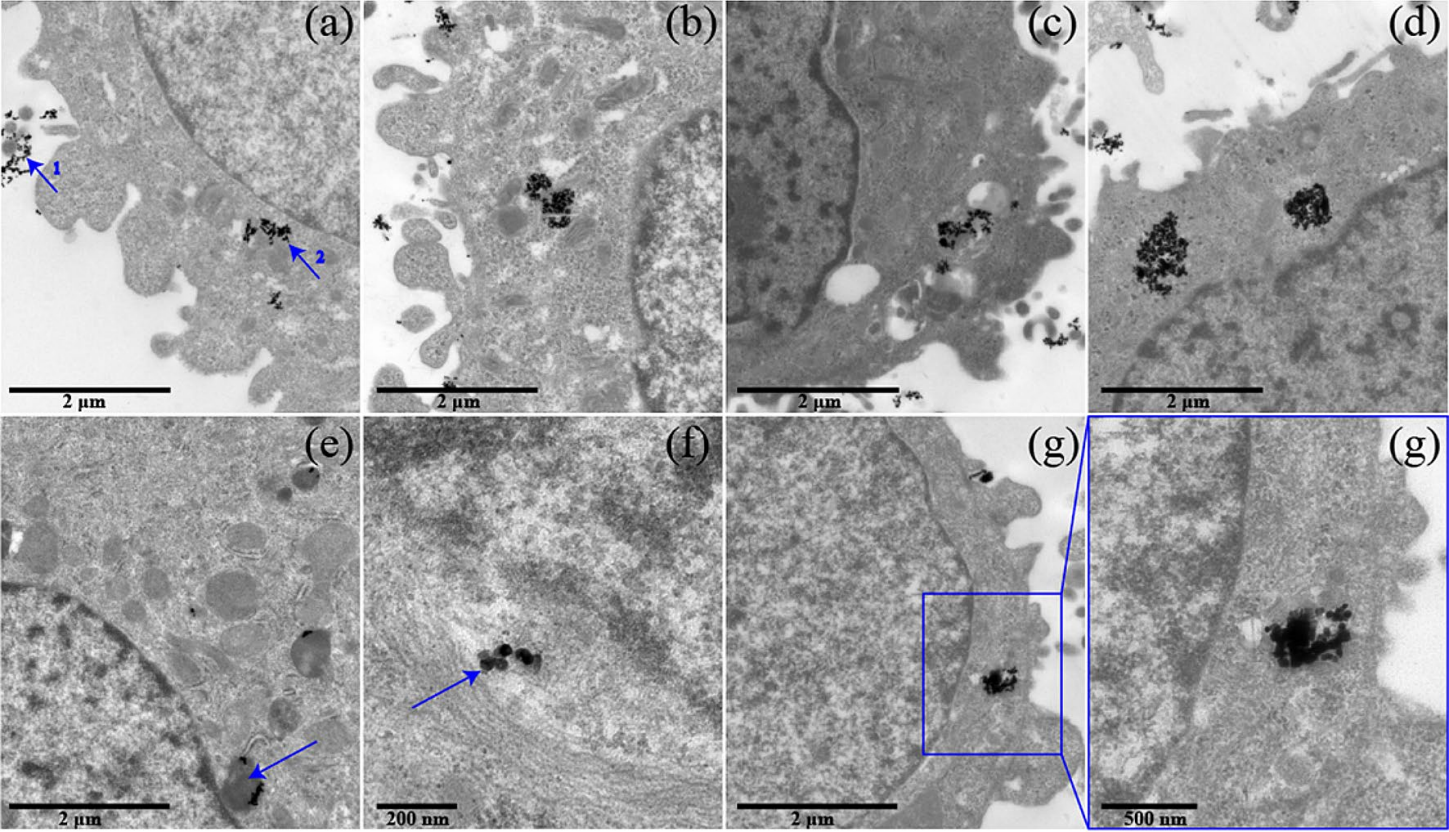
Localization in A549 cell of nAg_control_ (**a**), CA-nAg (**b**), FA-nAg (**c**) and TA-nAg (**d**) with incubated concentration of 75 μg ml^−1^, and CA-nAg (**e**) and TA-nAg (**g**) with incubated concentration of 10 μg ml^−1^. (**f**) CA-nAg in endoplasmic reticulum. 1 and 2 marked in (**a**) for surface associated AgNPs and intracellular AgNPs, respectively.

Figure 2 showed the distribution of intracellular NOMs-coated AgNPs in A549 cell. The intracellular NOMs-coated AgNPs were observed when exposing to NOMs-coated AgNPs (Fig. 2). When incubated concentration was 75 μg ml^−1^, the size clusters of intracellular CA- and TA-nAg were larger than them of nAg_control_ and FA-nAg (Fig. 2a–d). This suggested that CA- and TA-nAg would form clusters easily. When incubated concentration was 10 μg ml^−1^, the observed intracellular CA-nAg clusters were made up of a few particles. CA-nAg were observed in vesicles that was reached to nucleus (Fig. 2e) and endoplasmic reticulum where was much close to nucleus (Fig. 2f), while CA-nAg in nucleus was not found. The observed intracellular TA-nAg clusters at this concentration was comparably still in larger size (Fig. 2g). The surface associated AgNPs were observed in Fig. 2a, which implied that surface associated AgNPs were hard to remove with rinsing by PBS.

### Cellular uptake kinetics of NOMs-coated AgNPs by A549 cell

**Figure 3:**
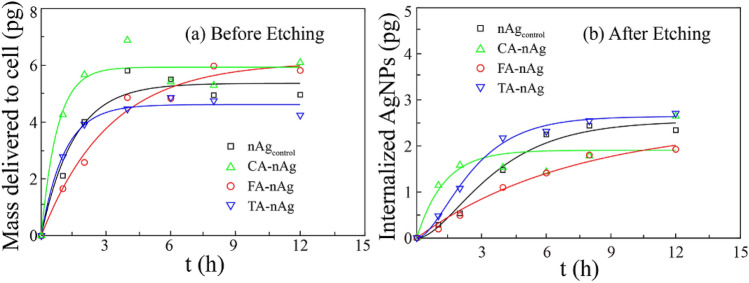
The internalization of AgNPs into A549 cell over 12 h after exposure with 10 μg ml^−1^ nAg_control_, CA-nAg, FA-nAg and TA-nAg in CM. (**a**) Cellular mass of AgNPs in A549 cell (*M*_*d*_, pg) and (**b**) Mass of AgNPs internalized to A549 cell (*M*_*i*_, pg) related to the incubation time for 0–12 h with. The colored solid lines were fitted with Langmuir adsorption model in (**a**) and internalizing model in (**b**) by Matlab 2016a.

Figure 3 showed time-dependent process of NOMs-coated AgNPs internalized into A549 cell. The internalizing process of nanoparticles can be described as two process^14,20,41^. Firstly, particles transport from CM to cell surface. This process is usually described as Langmuir absorption process which was depended the sites on cell surface to form a single layer due to association and dissociation. Secondly, surface associated particles are internalized into cell. Fig. S1 showed the schematic image of two process of kinetic mechanism. Langmuir absorption process expressed as follow^17,20,41^:

1 $$\frac{{dM_{d} }}{{dt}} = k_{a} C[M_{0} - M_{d} (t)] - k_{d} M_{d} (t)$$

where *M*_*d*_ is the mass of AgNPs delivered to single cell which is the sum of the mass of surface associated AgNPs^42^ and the mass of internalized AgNPs (*M*_*i*_), pg. *M*_*0*_ is the maximum capacity that the single cell surface could associate with AgNPs, pg. *C* is the concentration of AgNPs in CM, nM. *k*_*a*_ is the association factor, μM^−1^ h^−1^. *k*_*d*_ is the dissociation factor, h^−1^.

Solving the Eq. (1) got a time-dependent relationship of *M*_*d*_^20^:

2 $$M_{d} (t) = \frac{{k_{a} M_{0} C}}{{k_{a} C + k_{d} }}(1 - {\text{e}}^{{ - (k_{a} C + k_{d} )t}} )$$

The internalization process was expressed as follow^17,20,41^:

3 $$\frac{{dM_{i} }}{dt} = k_{i} M_{s} (t) - k_{out} M_{i} (t)$$

4 $$M_{d} (t) = M_{i} (t) + M_{s} (t)$$

where *k*_*i*_ and *k*_*out*_ are the rate constant for internalizing through cell membrane into cell via either endocytosis or other pathways and escaping from the cell via exocytosis or other pathways, respectively (h^−1^). *M*_*i*_ is the mass of surface associated AgNPs (pg).

The data of cellular uptake kinetics fitted with Eqs. (2) and (3) were shown in Fig. 3. Table 2 presented the fitting parameters of Langmuir adsorption model. The dissociation factor *k*_*d*_ of nAg_control_, CA-, FA- and TA-nAg was almost equal to 0. These implied that the dissociation process of AgNPs bound on A549 cell surface was weak. The larger the value of *k*_*a*_ is, the higher the affinity of AgNPs with cell surface will be^17^. The association factor—*k*_*a*_ of CA-nAg (1.006 μM^−1^ h^−1^) was similar to that of nAg_control_ (1.002 μM^−1^ h^−1^). However, *k*_*a*_ value of FA-nAg (352.2 μM^−1^ h^−1^) was much higher than that of nAg_control_ and the value of TA-nAg (1.386 μM^−1^ h^−1^) was slightly higher than that of nAg_control_. These implied that FA and TA absorbed on surface of AgNPs could increase the affinity of AgNPs with cell surface while CA affected very little.

**Table 2:** Fitting parameters for nAg_control_, CA-nAg, FA-nAg and TA-nAg bound to A549 cell with Langmuir adsorption model (incubated AgNPs concentration was 10 μg ml^−1^).

AgNPs	*M*_*0*_ (pg)	*k*_*a*_ (μM^−1^ h^−1^)	*k*_*d*_ (h^−1^)	R^2^
nAg_control_	5.31	1.006	0	0.95
CA-nAg	5.73	1.002	0	0.95
FA-nAg	6.10	352.2	0	0.98
TA-nAg	4.54	1.386	0	0.99

Table 3 presented the fitting parameters of internalizing model. The value of *k*_*i*_ and *k*_*out*_ of CA-nAg was about 5.82- and 11.3-folds higher than that of nAg_control_, respectively. The value of *k*_*i*_ and *k*_*out*_ of TA-nAg was about 1.69- and 1.14-folds higher than that of nAg_control_, respectively. To FA-nAg, *k*_*i*_ and *k*_*out*_ was much lower than them of nAg_control_ (around 0.29- and 0.38-folds to nAg_control_, respectively). Hence, the equilibrium time (*t*_*max*_) of internalization and exocytosis of CA- and TA-nAg was shorted from 18 h of nAg_control_ to 9 h and 13 h, respectively. The value of *t*_*max*_ of FA-nAg (52 h) was much larger than that of nAg_control_. The value of *M*_*imax*_ is frequently used in the evaluation of cellular uptake of nanoparticles with certain size or specific surface properties which could influence the association of nanoparticles on cell surface and the binding of nanoparticles with receptors^14,17,41^. Compared to nAg_control_ (2.54 pg per cell), *M*_*imax*_ of CA-nAg (1.84 pg per cell), FA-nAg (2.47 pg per cell) or TA-nAg (2.60 pg per cell) was closely related with the change of the value of *k*_*i*_/*k*_*out*_ (0.47, 0.7, 1.35 and 0.92, respectively) as shown in Table 3. Definitely, the parameter of *k*_*i*_/*k*_*out*_ indicated the value of dividing the rate of internalization by the rate of exocytosis. Accordingly, *k*_*i*_/*k*_*out*_ was suggested to be a valuable parameter to reflect cellular uptake of nanoparticles. Table 3 also shows that the values of k_out_ were greatly higher than those of k_i_ for nanoparticles except TA-nAg. It was reported that K_i_ values were less than K_out_ values for citrate- and PVA-coated Au nanospheres, while K_i_ and K_out_ values for PAA-coated Au nanospheres were significantly higher than those in the former two^20^. The authors explained that these were mainly caused by the different amount of Au nanospheres adsorbed onto the cell surface. These were consistent with our results that internalization process of coated nanoparticles would be influenced by the different organic ligands.

**Table 3:** Fitting parameters for nAg_control_, CA-nAg, FA-nAg and TA-nAg internalized into A549 cell with internalizing model (incubated AgNPs concentration was 10 μg ml^−1^).

AgNPs	*k*_*i*_ (pg h^−1^)	*k*_*out*_ (pg h^−1^)	*k*_*i*_/*k*_*out*_	*t*_*max*_ (h)	*M*_*imax*_ (pg)	R^2^
nAg_control_	0.198	0.216	0.92	18	2.54	1.0
CA-nAg	1.153	2.441	0.47	9	1.84	0.77
FA-nAg	0.057	0.082	0.70	52	2.47	0.97
TA-nAg	0.334	0.247	1.35	13	2.60	0.95

### Relationship of intracellular AgNPs with AgNPs delivering from suspension

**Figure 4:**
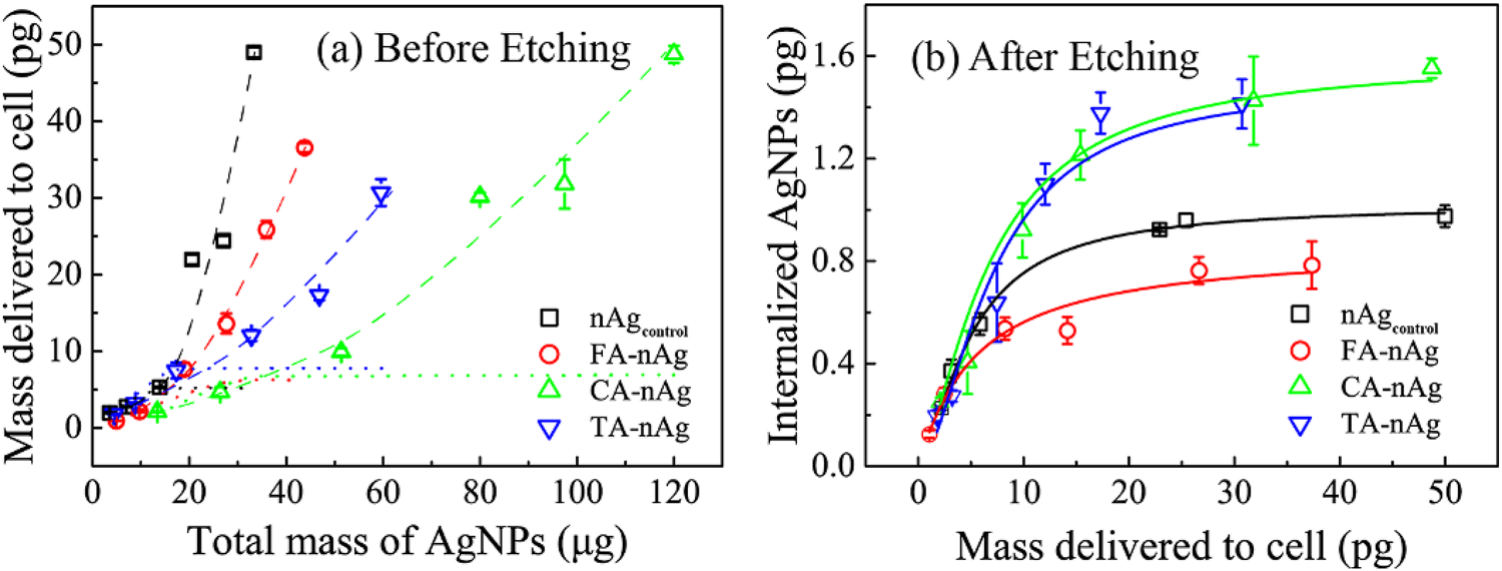
The internalization of AgNPs into A549 cell after exposure with different concentration of AgNPs suspension at 37 °C for 1 h. (**a**) Mass delivered to A549 cell (*M*_*d*_, pg) against to the increase of given mass (*M*_*T*_, μg) of nAg_control_, CA-nAg, FA-nAg and TA-nAg suspension. The dashed line was the trend line and the dotted line was the Langmuir rule line; (**b**) the mass of AgNPs internalized to A549 cell (*M*_*i*_, pg) against to *M*_*d*_. The solid color lines were fitted based on logistics model.

Figure 4 presented the relationship of *M*_*i*_ with the given mass of AgNPs in total (*M*_*T*_) and *M*_*d*_. With the increase of *M*_*T*_, *M*_*d*_ exponentially increased as shown in Fig. 4a. This was different from Langmuir adsorption, since the aggregation and sedimentation happened in concentrated AgNPs suspension. Aggregation and sedimentation enhanced the delivering process of AgNPs onto cell surface and broke the rule of Langmuir association process. As a result, *M*_*d*_ of concentrated AgNPs suspension depended on stability of AgNPs. To reach the same level of *M*_*d*_, CA-, FA- and TA-nAg need more given mass in CM than nAg_control_ (such as to get 20 pg per cell of *M*_*d*_, need *M*_*T*_ 20, 70, 50 and 30 μg, respectively to nAg_control_, CA-, FA- and TA-nAg). This meant that CA-, FA-and TA-nAg were more stable in CM than nAg_control_. It was consistent with the results in Fig. 1d.

As shown in Fig. 4b, the relationship of *M*_*i*_ and *M*_*d*_ was likely subject to Logistic model which was found to fit well with the uptake of magnetic iron nanoparticles by T98G and U251 cell^19^. The Logistic model was expressed as follow^19^:

5 $$M_{i} = M_{imaxC} - \frac{{M_{imaxC} }}{{1 + ({{M_{d} } \mathord{\left/ {\vphantom {{M_{d} } {EC_{50} }}} \right. \kern-\nulldelimiterspace} {EC_{50} }})^{p} }},$$

where *M*_*imaxC*_ was maximum value of *M*_*i*_, pg; *EC*_*50*_ was *M*_*d*_ for 50% of *M*_*imaxC*_, pg; *p* was the slope factor^19^.

Table 4 presented the fitting parameters of Logistic model. *M*_*imaxC*_ of CA-nAg and TA-nAg was 1.59 and 1.50 pg, respectively, higher than that of nAg_control_ (1.02 pg). *M*_*imaxC*_ of FA-nAg was 0.85 pg, less than that of nAg_control_. *M*_*0C*_ of CA-nAg and TA-nAg was 6.78 and 5.39 pg, much higher than that of nAg_control_ (5.00 pg). *M*_*0C*_ of FA-nAg (5.39 pg) was slightly higher than that of nAg_control_.

**Table 4:** Fitting parameters for nAg_control_, CA-nAg, FA-nAg and TA-nAg internalized to A549 cell based on logistic model (incubated AgNPs concentration were 0–60 μg ml^−1^).

AgNPs	*M*_*imaxC*_ (pg)	*EC*_50_ or *M*_*0C*_ (pg)	*p*	*R*_*T*_^a^	R^2^
nAg_control_	1.02	5.00	1.51	20.4	1.0
CA-nAg	1.59	6.78	1.45	23.5	0.99
FA-nAg	0.85	5.39	1.04	15.8	0.97
TA-nAg	1.50	7.06	1.68	21.2	0.95

^a^*R*_*T*_ = *M*_*imaxC*_/*M*_*0C*_ × 100%, was defined to reflect the efficiency of surface association AgNPs transferring to internalized AgNPs.

### Cellular uptake pathway for NOMs-coated AgNPs into A549 cell

**Figure 5:**
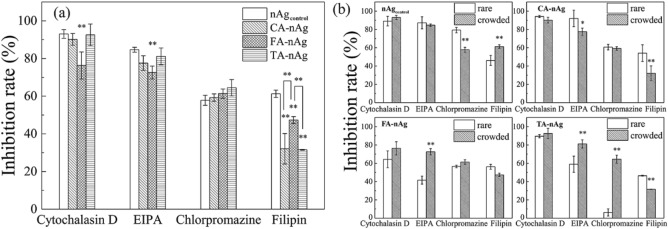
The inhibition rate (inhibition rate = (1-*M*_*i with inhibitor*_/*M*_*i without inhibitor*_) × 100%) of cytochalasin D (5 μM), EIPA (5 μM), chlorpromazine (30 μM) and filipin (0.5 μg ml^−1^) for (**a**) the difference among nAg_control_, CA-nAg, FA-nAg and TA-nAg internalizing to “crowded” state cells, and (**b**) the difference between “rare” state cells and “crowded” state cells. (The significant difference was signed with *(p < 0.05) and **(p < 0.01)).

Figure 5a presented the influences of inhibitors on the internalization of AgNPs under “crowded state” cells and their influences between “rare” state cells and “crowded” state cells were show in Fig. 5b. These inhibitors (cytochalasin D, EIPA, chlorpromazine and filipin) have few influences to the stability of AgNPs suspension and little decrease in A549 cell viability incubated for 1 h under the used concentrations as shown in Fig. S2. To “crowded state” cells, the inhibition rate of internalization of AgNPs by cytochalasin D was at least 90%, except for FA-nAg (76%) (Fig. 5a). The addition of EIPA also resulted in significant inhibition of internalization of the positive control (nAg_control_), CA-, FA- and TA-nAg at the rate of 85, 76, 73 and 81%, respectively. Chlorpromazine have also caused a large extent of decrease in internalization of nAg_control_, CA-, FA- and TA-nAg with the rate of 58, 59, 61 and 64%, respectively. The inhibition rate of internalization of nAg_control_ by filipin was 61%, significantly higher than filipin for CA-, FA- and TA-nAg with the rate of 32, 47 and 31%, respectively. The influences of inhibitors to “rare state” and “crowded state” cells were different (Fig. 5b). The inhibition rate for “rare state” cells was significantly lower than that for “crowded state” cells, including EIPA to FA- and TA-nAg, chlorpromazine to TA-nAg, and filipin to nAg_control_ (Fig. 5b). However, some reverse phenomena were observed, including EIPA to CA-nAg, chlorpromazine to nAg_control_, and filipin to CA-nAg and TA-nAg (Fig. 5b).

## Discussion

The size of FA-nAg was little changed as shown in Fig. 1a. Table 1 showed that carbon content of FA-nAg was 2.64%, higher than that of nAg_control_ (1.77%). This suggested that FA had been absorbed on the surface of AgNPs. FA-nAg presented more negative value of zeta potential in water (− 42.7 mV) than nAg_control_ (− 24.2 mV) (Table 1). Moreover, FA could also lightly enhance D_H_ of FA-nAg in CM comparing to nAg_control_ in CM (121 nm to 117 nm in average) as shown in Table 1. However, carbon content of TA-nAg (1.47%) was lower than that of nAg_control_ (Table 1). This suggested that TA decreased the amounts of PVP coated on AgNPs. Moreover, C:N of TA-nAg was much higher than that of nAg_control_ (7.12 to 5.1). This suggested that TA had absorbed on surface of AgNPs replacing parts of coated PVP. D_H_ of TA-nAg in CM was much lower than that of nAg_control_ in CM (81 nm to 117 nm in average) for shrinking hydration layer in water solution (Table 1). D_H_ of CA-, FA- and TA-nAg in CM kept steady within 30 min as nAg_control_ in CM, implied that little aggregation happened in dilute AgNPs suspension (1 μg ml^−1^). However, red-shift for 2 nm observed in the SPR spectrum of CA-, FA- and TA-nAg as nAg_control_ suggested that light aggregation happened in concentrated AgNPs suspension (20 μg ml^−1^)^43,44^. The concentrated suspension of CA-, FA- and TA-nAg in CM seemed to be more stability than nAg_control_ since only 8.4, 6.6 and 7.0% decreased in A_max_ of them, lower than 15.6% of nAg_control_.

The much higher affinity of FA-nAg to A549 cell than others were found as shown in Table 2. Cho et al. found that poly(allyamine hydrochloride) or PAA coated AuNPs showed higher affinity to SK-BR-3 breast cancer cells than others (k_a_ was 10 times to others) because of the positive charge of amino function group^20^. FA were comprised of aromatic, carboxylic acid and amino function group according to the characterization in our previous report^37^. Accordingly, the amino function group in FA could make the difference.

The value of *k*_*i*_ reflect the rate of particles internalizing into cell^17,20,41^. Table 3 showed that value of k_i_ of CA-nAg and TA-nAg was larger than that of nAg_control_. Harris et al. proved that hydrating layer hinders protein adsorption and subsequent internalization^45^. Table 1 showed that the thickness of hydrating layer of CA-nAg and TA-nAg was lower than that of nAg_control_ (D_H_ of them was 99, 81 and 117 nm, respectively). Therefore, the increase of rate of CA-nAg and TA-nAg internalizing into cell were related to the decrease of hydrating layer of these AgNPs. However, the value of *k*_*i*_ of FA-nAg was still much lower than that of nAg_control_ even though the similar D_H_ (121 nm) with nAg_control_ as shown in Tables 1 and 3.

Table 5 recorded the *M*_*i*_ of AgNPs and AuNPs into cells that have reported in many literatures^8,20,33,46–50^. *M*_*i*_ of these AgNPs into cancer cells were about 2.1 to 10 pg at a same order of magnitude with the results in this study, despite of different size, surface functionalization or other experimental conditions^8,47,50^, which showed much difference from the normal cells (*M*_*i*_ = 47 pg for Pk 15 cells)^48^. Comparing with the *M*_*i*_ of AgNPs into cancer cells, *M*_*i*_ of AuNPs showed smaller value of about 0.12 to 1.23 pg (about 0.07 to 0.67 pg after converting into density of AgNPs)^20,46,49^. Cho et al.^20^ reported the kinetics of CA-nAu internalizing into SK-BR-3 cells wherein *k*_*i*_ of CA-nAu was about 3.3 × 10^–5^ pg h^−1^, which was far below *k*_*i*_ of CA-nAg in this study, consequently the equilibrium of CA-nAu into SK-BR-3 cells were not observed during 24 h of experiment, which could explain that the reported *M*_*i*_ of AuNPs lower than *M*_*i*_ of AgNPs.

**Table 5:** Summarizing of *M*_*i*_ of AgNPs and AuNPs in reported literature.

Nanoparticles (sphere)		Incubation			Removal method for cell surface associate nanoparticles	*M*_*i*_ (per cell)	References
Particles	Size (nm)	Cell Strain	Concentration	Time (h)			
**Silver (density: 10.5 g cm**^**−3**^**)**			μg ml^−1^			pg	
nAg_control_	27.9 ± 7.9	A549 cells	10	t_max_	etching with K_3_Fe(CN)_6_-Na_2_S_2_O_3_	5.31/2.54^a^	This study
CA-nAg	25.1 ± 6.3					5.73/1.84^a^	
FA-nAg	27.2 ± 7.0					6.10/2.47^a^	
TA-nAg	27.8 ± 6.2					4.54/2.60^a^	
PVP-nAg	10	BEAS-2B cells	10	4	rinsed with PBS for 5 times	2.1	^8^
CA-nAg	10					2.9	
	75					3.2	
Naked nAg	50					10	
PVP-nAg	12.4	HepG2 cells	10	24	rinsed with PBS for several times	6.8	^50^
nAg	20–200	A549 cells	10	4	rinsed with PBS for 2 times	4.5^b^	^47^
CA-nAg	N/A	Pk15 cells	10	24	rinsed with PBS for several times	47	^48^
PVP-nAg	70 ± 25	HMSC	10	24	TEM sections + image analysis	20% of NPs in TEM sections^b^	^33^
**Gold (density: 19.3 g cm**^**−3**^**)**			nM				
CA-nAu	15	Hela cells	10	8	rinsed with PBS for 3 times	1.23^a^	^49^
CA-nAu	15	A549 cells	20	4	TEM sections + image analysis	0.98	^46^
PEG-nAu						0.29	
CA-nAu	17.7	SK-BR-3 cells	0.027	24	etching with I_2_-KI	0.33/0.24^a,b^	^20^
PVA-nAu						0.15/0.12^a,b^	

*N/A* no data, *PEG* polyethylene glycol, *PVA* polyvinyl alcohol.

^a^Before/after etching.

^b^Data acquired from scaling the Figures panel in related literature.

According to the Logistic model, *M*_*0C*_ of nAg_control_ and FA-nAg were close to the value of *M*_*0*_ (Table 2). However, *M*_*0C*_ of CA- and TA-nAg were much higher than that of *M*_*0*_. These implied that when suspension of CA- and TA-nAg come to concentrate, the associated particles could gather together tightly and form large cluster on cell surface due to their small size of D_H_. The clusters made higher value of appearance *M*_*0*_ that was called “*M*_*0C*_”. As a consequence, *M*_*imaxC*_ of CA- and TA-nAg was higher than that of nAg_control_ which was different from the situation of *M*_*imax*_. Ratio of internalized AgNPs transferring from *M*_*0C*_ (*R*_*T*_, *R*_*T*_ = *M*_*imaxC*_/*M*_*0C*_ × 100%) was defined to reflect the efficiency of surface association AgNPs transferring to internalized AgNPs. *R*_*T*_ of CA-nAg and TA-nAg (23.5% and 21.2%) were higher than *R*_*T*_ of nAg_control_ (20.4%), and *R*_*T*_ of FA-nAg (15.8%) was lowest. Combined with the conclusion of *k*_*i*_ (CA-nAg, TA-nAg > nAg_control_ > FA-nAg), these implied that CA and TA-nAg showed stronger ability but FA-nAg presented weaker ability of transport across plasma membrane than nAg_control_.

Cytochalasin D is a cell permeable toxin which can disrupt actin filaments^51^. EIPA is a Na^+^/H^+^ ion channel blocking agent that inhibits the macropinocytosis-mediated pathway^29,52^. Chlorpromazine can prevent from the formation of clathrin in cells and is used to depress the uptake pathway of CME^53,54^. The addition of these inhibitors resulted in the suppression of internalization of the pristine AgNPs–nAg_control_, indicated that the uptake pathway of AgNPs were mostly depended on actin and contributed a lot to both macropinocytosis and CME. Moreover, the inhibition rate of these inhibitors to NOMs-coated AgNPs was less affected by CA, FA and TA, while the inhibition rate of cytochalasin D and EIPA to FA-nAg were significantly less than them to the positive control—nAg_control_ (Fig. 5a). Accordingly, actin-involved uptake pathway or macropinocytosis would have less contribution to uptake of FA-nAg than uptake of the pristine AgNPs–nAg_control_. It could be the reason of lower efficiency in uptake for FA-nAg than for nAg_control_ (*k*_*i*_ and *k*_*out*_ of FA-nAg were far below them of the pristine AgNPs–nAg_control_ as presented in Table 3).

**Figure 6:**
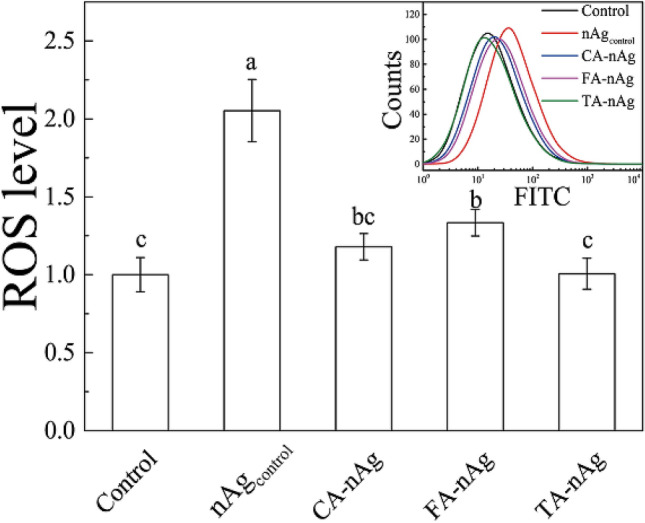
ROS level of A549 cell after incubated with 75 μg ml^−1^ pristine nAg, CA-nAg, FA-nAg and TA-nAg for 24 h (The significant difference were signed with different letters).

Filipin, a drug can bind with sterol, is known as an inhibitor of LME^30,55^. The addition of filipin resulted in 61% decrease in the internalization of the pristine AgNP–nAg_control_. This indicated that LME was also involved in the internalization of AgNPs. However, inhibition rate of CA-nAg, FA-nAg and TA-nAg by filipin was 32, 47 and 31%, respectively that were consistently lower than the rate to the pristine AgNPs–nAg_control_ (61%). These indicated that CA, FA or TA would change the way of uptake of AgNPs mainly through depressing the contribution of LME. The results were highly accordance with yielded ROS level by these AgNPs that nAg_control_ could arouse ROS level for twofold but 1.3-fold for FA-nAg and no significant influence for CA-nAg and TA-nAg (Fig. 6). LME is a unique pathway for nanoparticles that the intracellular vesicle could escape from lysosomal and uptake the nanoparticles into cytoplasm^26,28^. Huk et al. found that AgNPs caused much higher level of cytotoxicity since it could have reached into nucleus and mitochondria^4^. It implied that the higher dependence on LME of AgNPs would have higher opportunity to reach into nucleus or mitochondria and cause more damage to cells.

**Figure 7:**
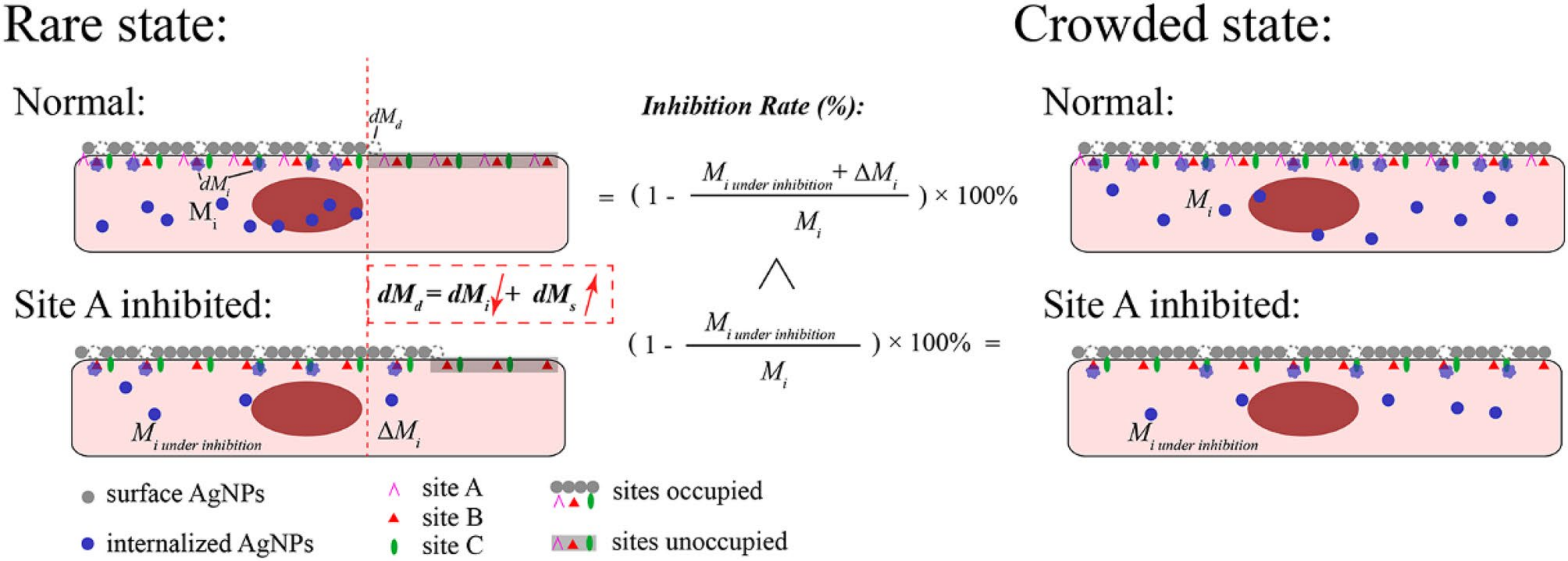
Schedule of the process of AgNPs entering normal A549 cell and the cell with site A inhibited to illuminate the difference of inhibition rate between “rare” state and “crowded” state.

Figure 7 illuminated the reason about lower inhibition rate of “rare” state than “crowded” state. The “rare state” means that mass of cell surface associated AgNPs^42^ were far below *M*_0_ while “crowded state” means that mass of associated AgNPs were enough to reach *M*_0_. To the addition of inhibitor to “rare state” cells, the *M*_*s*_ would be higher than M_s_ without inhibitors, because *M*_*i*_ would be reduced but *M*_*d*_ might be rarely impacted according to Eq. (4). Thus, more free sites on cell surface would be occupied and result in exceeding *M*_*i*_. Therefore, the calculated inhibition rate would be lower than what it should be. This may be called as the “waning and waxing” phenomenon. To the “crowded state”, this phenomenon could be ignored since *M*_*s*_ was a constant.

However, some reverse phenomenon happened because of certain properties of endocytosis pathway. LME inhibited by filipin is known to be a receptor-specific uptake and usually form 50 to 80 nm caveolae in size^26,28^. As shown in Table 4, it can be concluded that CA-nAg and TA-nAg could form clusters on cell surface under concentrated CA-nAg and TA-nAg suspensions. Accordingly, the formed clusters were not appropriate in size to be trapped by caveolae. The inhibition rate of filipin to “crowded state” cell of CA-nAg and TA-nAg was less than the inhibition rate to their “rare state” cell.

In summary, CA treatment reduced D_H_ and enhanced the colloidal stability of CA-nAg in CM comparing to the pristine AgNPs–nAg_control_. Consequently, the increase for both of *k*_*i*_ and *k*_*out*_ but decrease in *M*_*i*_ were found. TA treatment reduced D_H_ and enhanced colloidal stability of TA-nAg in CM compared to the pristine AgNPs–nAg_control_. Consequently, the increase for *k*_*i*_, *k*_*out*_ and *M*_*i*_ were found. FA enhanced the stability of FA-nAg in CM, but much decrease for *k*_*i*_, *k*_*out*_ and *M*_*i*_ were found which resulted from less dependent on actin involved uptake pathway and macropinocytosis than the pristine AgNPs–nAg_control_. In addition, intracellular mass of these AgNPs were dependent on *M*_*d*_, which obeyed Logistic model. According to the internalization model and Logistic model, CA and TA-nAg showed stronger ability but FA-nAg presented weaker ability of transport across plasma membrane than the pristine AgNPs–nAg_control_. Moreover, uptake of CA-, TA- and FA-nAg was less dependent on LME comparing to the pristine AgNPs–nAg_control_, which resulted from cell surface association state of AgNPs that affected by NOM.

## Methods

### Natural organic matters

The NOM used in this study include CA, TA and FA. CA and TA were purchased from Sinopharm Chemical Reagent Co., LTD. FA was extracted from the sediments of Xuanwu Lake at Nanjing, China, and the properties were presented in our previous report^37^.

### Preparation and characterization of NOM coated nAg

The pristine silver nanoparticles of 20 nm were synthesized according to the previous reports with minor modification (more details seen the Supporting Information, SI)^56^. The obtained silver was marked as p-nAg for the subsequent treatment.

NOMs-treated nAg was made according to our previous study^37^. Briefly, the obtained p-nAg suspension was treated with solutions containing citric acid, tartaric acid and fulvic acid Concentrations of CA and TA in solution were set as 10 mM and FA were 200 mg l^−1^ (more details seen the Supporting Information, SI).

The sizes of the pristine and NOM-coated AgNPs were characterized by Transmission Electron Microscope (TEM, JEM-2100 (HR), Japanese JEOL Corporation). The carbon and nitrogen contents of these samples were determined using element analyser (EA, CHN-O-Rapid, Germany Heraeus Corporation). The stability of the pristine and NOM-coated AgNPs suspensions in Ham’s F-12K (Kaighn’s) Medium (1×, Gibco) which is used as grow up medium (CM) for A549 cell in this study supported with 1% fetal bovine serum (FBS, Hyclone) and 1% antibiotics (penicillin streptomycin sol, Gibco) were characterized by UV–Vis spectrometer (UH5300, Japanese Hitachi Corporation) at 37 °C. Briefly, 5 mg of the pristine and NOM-coated AgNPs was put in 5 ml 1% FBS supported CM. The suspensions were diluted with 1% FBS supported CM to a final concentration of 20 μg ml^−1^. The absorbance from 300 to 600 nm (step by 2 nm) of suspensions were detected at 0, 10, 20, 30, 40, 60 min, respectively. Hydrodynamic diameter (D_H_, nm) and zeta potential value (ZP, mV) of the suspensions (diluted to 1 μg ml^−1^) were monitored over 30 min at 37 °C by nanoparticle size analyzer (90Plus, Brookhaven Instruments Corporation). The initial D_H_ was determined based on the average value of dynamic light scattering (DLS) data within 3 min.

### Cell culture

Ham’s F-12K (Kaighn’s) Medium (1×, Gibco) was used as culture medium for A549 epithelial cells after adding with 1% antibiotics (penicillin streptomycin sol, Gibco). A549 cells were cultured in 10% FBS supported CM. The cultures were incubated at incubator (37 °C, 5% CO_2_) and the medium was changed every two days.

### Etching AgNPs bound on the cell surface

Etching method was proposed and verified by Gray B. Braun for removing the absorbed the pristine and NOM-coated AgNPs on cell surface which disrupt the quantitative of intracellular AgNPs^21^. Actually, etching method failed to clean the well-plate touching side of adherent cell where solvent was hard to infiltrate while AgNPs could be transferred from top side surface due to the fluidity of cell membrane. An etching method integrated adherent and suspended cell to remove the absorbed AgNPs on cell surface for the quantitative analysis of the intracellular AgNPs was developed and the related experiments with results and discussion were described in SI. The low cytotoxicity of our used etchants to A549 cell in short etching time was shown in Fig. S3a. The high efficiency of the etching method to remove association AgNPs on A549 cell surface was also proved in Fig. S3b. Therefore, based on the quantitative analyses of particles internalized into cell.

### Kinetics of the pristine and NOM-coated AgNPs uptake by A549 cell

A549 cells were seeded in 12 well-plate for 24 h prior to exposure with the pristine and NOM-coated AgNPs. At the following day, the CM was removed, and then rinsed with PBS for twice. After the addition of 10 μg ml^−1^ AgNPs suspension, cells were incubated for 0, 1, 2, 4, 6, 8 and 12 h, and then treated with the etchant and collected.

### Relationship of intracellular AgNPs with AgNPs delivering from suspension

After incubation for 24 h of seeded cells in 12 well-plate, the CM were removed and cells were rinsed with PBS for twice. Designed concentrations of AgNPs suspension were added and cells were incubated for 1 h. Cells were treated with the etchant and collected. Concentrations of AgNPs were set as: 0–35 μg ml^−1^ for nAg_control_, 0–50 μg ml^−1^ for CA-nAg, 0–40 μg ml^−1^ for FA-nAg and 0–60 μg ml^−1^ for TA-nAg. Fig. S4 showed that cell viability of AgNPs was above 80% even the incubated concentration of AgNPs up to 100 μg ml^−1^, which implied that concentration of AgNPs used in this experiment were harmless to A549 cell.

### Cellular uptake pathway of NOM-coated AgNPs on A549 cell

Some literatures report that cellular uptake of particles are dependent on their aggregation^32^ or aggregation behavior^57^ on cell surface. Therefore, the gathering state of AgNPs on cell surface would affect the cellular uptake pathway utilized by AgNPs. Accordingly, cellular uptake pathway for NOM-coated AgNPs to A549 cell were studied under two levels of AgNPs density on cell surface (rare state and crowded state).

#### Rare state

Rare state means the level of AgNPs associated with cell surface was much less than maximum capacity to accept AgNPs on A549 cell surface. Firstly, A549 cells were seeded in 12 well-plate. The cells were pretreated with inhibitors (their final concentrations were 5 μM for cytochalasin D, 5 μM for EIPA, 30 μM for chlorpromazine and 0.5 μg ml^−1^ for filipin, respectively) for 30 min in incubator. Then, cells were rinsed with PBS for 1 time and exposure to 10 μg ml^−1^ of pristine or NOMs-coated AgNPs suspension with inhibitor (kept the same concentration), and incubated for 1 h in incubator. Finally, cells were treated with etching method and collected.

#### Crowded state

Crowded state means the mass of AgNPs associated with cell surface was enough to reach maximum capacity to accept AgNPs on A549 cell surface. Similar to above, after pretreated with inhibitor, cells were rinsed with PBS for 1 time and exposure to pristine or NOMs-coated AgNPs suspension with inhibitor. To make 20 pg per cell AgNPs associated on cell surface before cellular uptake starting, cells were firstly incubated for 1 h at 4 °C. Then, cells were put in incubator for next 1 h. Finally, cells were treated with etching method and collected. Final concentration of nAg_control_, CA-nAg, FA-nAg and TA-nAg for “crowded state” were 20, 70, 50 and 30 μg ml^−1^, respectively.

Cells exposed to the pristine silver nanoparticles with inhibitor were set as positive control (marked as nAg_control_). Cells exposed to the pristine and NOMs-coated AgNPs without any inhibitor were set as negative control. Cells incubated with inhibitor and without pristine and NOMs-coated AgNPs were set as blank control. The experiment was run with triplicate.

### TEM observation of intracellular pristine and NOM-coated AgNPs

TEM has frequently been used to observe the localization of nanoparticles^8,14^. A549 cells were centrifuged and rinsed by PBS after 6 h exposure to 75 μg ml^−1^ nAg_control_, CA-nAg, FA-nAg and TA-nAg or 10 μg ml^−1^ CA-nAg and TA-nAg. The harvested cells were prefixed in 2.5% glutaraldehyde at 4 °C overnight and washed with PBS three times. Subsequently, the cells were stained with 1% osmic acid followed by gradient dehydration with ethanol and acetone. Then, the samples were embedded in epoxy resin, sectioned, and post stained with lead citrate and uranyl acetate before TEM observation. Finally, cells were observed using the TEM.

### ROS level

The cells were seeded in 12 well-plate for 24 h prior to exposure with AgNPs. Seeding density was 5 × 10^5^ cells per well. Cells were exposure to 1 ml 75 μg ml^−1^ AgNPs suspension for 24 h. Then, the plates were rinsed with PBS for twice and loaded with 10 μM DCFH-DA in CM for 20 min in incubator. Thereafter, cells were rinsed with CM for three time and treated with 0.2 ml EDTA-trypsin solution. The suspended cells were collected with PBS and the fluorescence was recorded with a flow cytometer (MoFlo XDP, Beckman Coulter) by reading 5 × 10^4^ cells at FL1 channel (excitation 485 nm, emission 535 nm).

### Elemental analysis

The cells (cell number: 5 × 10^5^) were digested with concentrated HNO_3_. Concentrations of Ag were measured using an inductively coupled plasma optical emission spectrometry (ICP-OES, Optima 5300, Perkin-Elmer SCIEX, USA). The calibration standard solutions were diluted from obtained by the dilution of the standard stock solutions (Custom Assurance Standard) purchased from SPEX CertiPreP (1000 mg l^−1^, Lot number: 28-232CR) with 2% HNO_3_ (V/V). The relative percentage differences of parallel samples were within 20%, or the experiments were repeated.

### Data analysis

The significant differences were analysed by independent-sample T tests in SPSS statistic 17.0. First-order removal model and Logistic model were fitted with the trends of AgNPs sedimentation in CM and the mass reliable internalization process, respectively in origin 9.1. Langmuir absorption model and internalizing kinetic model were fitted with cell surface association process and internalizing kinetic of AgNPs, respectively, in Matlab R2016a.

## Supplementary Information

Supplementary Information.
