# Supplementary material for: Transport of environmental natural organic matter coated silver nanoparticle across cell membrane based on membrane etching treatment and inhibitors
Source: Sci Rep. 2021 Jan 12;11:507. doi: 10.1038/s41598-020-79901-y (PMC7803783; doi:10.1038/s41598-020-79901-y)
Supplement: Supplementary file 1 — Supplementary Information. [file 41598_2020_79901_MOESM1_ESM.docx]

Supplementary materials

Transport of environmental natural organic matter coated silver nanoparticle across cell membrane based on membrane etching treatment and inhibitors

Laijin Zhong^a^, Sisi Chen^a^, Zhijie Tang^a^, Xuewen Guo^a^, Xin Hu^a*^, Weijuan Zheng^b^, Hong-zhen Lian^a*^

^a^ State Key Laboratory of Analytical Chemistry for Life Science, School of Chemistry & Chemical Engineering and Centre of Materials Analysis, Nanjing University, Nanjing 210023, PR China

^b^ State Key Laboratory of Pharmaceutical Biotechnology, School of Life Science, Nanjing University, Nanjing 210023, PR China

Corresponding authors: *Xin Hu. Phone: +86-25-83592247; e-mail: huxin@nju.edu.cn; *Hong-zhen Lian. Phone: +86-25-89681929; e-mail: hzlian@nju.edu.cn.

***Synthesis of 20 nm silver nanoparticle***

The method was based on the previous reports with a few modification. 20 g PVP (Mw = 10000) was slowly added in 150 ml ethylene glycol at room temperature with continuous stirring until PVP was thoroughly dissolved. 800 mg AgNO_3_ was then slowly added at room temperature with continuous stirring until AgNO_3_ was complete dissolved. Then, the system was heated up to 120 °C at a constant rate of 3 °C min^-1^. The reaction was allowed to maintain for 1 h at 120 °C. At the end, the colloidal dispersion was cooled down to the room temperature in a water bath. With 500 ml acetone added in, the silver was separated from the solution by centrifugation at 15000 rpm for 30 min. The precipitates was washed with pure water for 3 times to remove the fragilely combined PVP on silver surface and separated by centrifugation at 15000 rpm for 30 min. The obtained silver was dispersed in 10 ml pure water for the subsequent treatment.

***NOMs treated nAg***

Briefly, a certain volume of silver suspension were mixed with 50 ml solution with and without NOMs in a conical flask stirring with magnetic for 2 days kept in the dark place to make the final concentration of 2 μg ml^-1^ Ag. The suspensions were centrifuged with 8960 g for 10 minutes. The precipitates were freeze-dried, grinded, and stored for future experiments. NOM solutions were obtained by dissolving solid FA, CA or TA in 30 mM NaNO_3_ solution and adjusting to pH 7. Concentrations of CA and TA in solution were set as 10 mM and FA were 200 mg l^-1^. The ultrapure water in this study were taken from Milli-Q Ultra Purified Water System.

**Cell viability test of etchant.**

A549 cells were seeded in 96 well-plate 24 h prior to exposure with etchant. Seeding density was 10^4^ cells per well in 200 μl CM. At the following day, cells were exposed to 100 μl etchant supported with 1% FBS for 3, 5, 10, 20 and 30 min, and 100 μl etchant without FBS for 20 min. After rinsing with PBS for three time, the plates were added in 100 μl PBS and 10 μl CCK-8 reagent per well and then incubated for 20 min. The absorbance was measured by spectrophotometry at 450 nm. These experiments were repeated on six occasions.

The results presented in Fig. S3a. The results showed that the A549 cell viability was still maintained over 80% as contacting etchant with 1% FBS up to 20 min. When contacted for 20 min, the A549 cell viability started to decrease significantly. If the contacted time over 30 min, cell viability dropped to 78%. A549 cell contacted with etchant without FBS for 20 min, the cell viability decreased to 74.7% and significantly lower than cell viability using etchant with 1% FBS.

The etchant used by Gray B. Braun was FBS free solution. Our experiment proved that the addition of 1% FBS would significantly decrease the cytotoxicity of etchant, which could make the etchant more practical. Etchant supported with 1% FBS caused no influence to A549 cell viability in short time. We suggested that the contacting time should better be controlled below 10 min.

**Improvement and effective of etching method.**

***Adherent etching process (AE):*** The cells after incubated with AgNPs for 4 h were treated with 0.5 ml etchant (phosphate buffer saline (PBS) solution with 10 mM K_3_Fe(CN)_6_ and 10 mM Na_2_S_2_O_3_, supported with 1% FBS) for 3 minutes, and repeated for twice.

***Suspension etching process (SE):*** The cells after incubated with AgNPs for 1 or 4 h were treated with 0.5 ml etchant (phosphate buffer saline (PBS) solution with 10 mM K_3_Fe(CN)_6_ and 10 mM Na_2_S_2_O_3_, supported with 1% FBS) for 3 minutes. Then, the adherent cells were washed twice with PBS and treated with 0.2 ml EDTA-trypsin solution (w, % = 2.5%). The cells were suspended with 0.5 ml etchant for 1 minutes, and then collected from removing the supernatant by centrifuging at 1000 rpm, 4 °C. And repeat for once.

The whole etching process were carried out at 4 °C to prevent cells from impairing. The etched solution were collected. Concentration of Ag were detected after sample treatment.

The results were presented in Fig. S3b. The results showed that AE for three time made AgNPs removing for 93.1, 6.1, and 0.8% from the cells incubated with AgNPs for 4 h, respectively. The SE for three time made AgNPs removing for 73.8, 25.6 and 0.6% from the cells incubated with AgNPs for 4 h and removing for 90.6, 9.2 and 0.2% from the cells incubated with AgNPs for 1 h, respectively.

Considering the same first etching process of AE and SE but larger first etching ratio of AE (R_A1_) than first etching ratio of SE (R_S1_), the total etching mass of SE (SE_total_) was 1.3 fold to the total etching mass of AE (AE_total_) based on the same first etching mass (AE_total_ × R_A1_=SE_Total_ × R_S1_). It proved that SE was more effective than AE of removing AgNPs from cell surface. And the increase of etching mass mainly came from the second etching process of SE which was the first suspending etching process, since suspending cells exposed to etchant with the bottom side that attached to plate and hard to contact with etchant. AgNPs associated with the bottom side resulted from the fluidity of cell membrane. The second etching ratio of SE for 4 h cells (R_S2-4_) was larger than the second etching ratio of SE for 1 h cells (R_S2-1_). It implied that AgNPs associated with the bottom side would increase as the incubation time. The third etching ratio of SE was extremely low, which suggested that the associated AgNPs on cell surface was mostly removed after the second etching process of SE. We recommended to remove AgNPs attached on adherent cells with SE (the third process could be cancelled to decrease impairing degree to cells), especially as incubation for a long time.

***Improved etching method:*** The cells after incubated with AgNPs were treated with 0.5 ml etchant (phosphate buffer saline (PBS, Hyclone) solution with 10 mM K_3_Fe(CN)_6_ and 10 mM Na_2_S_2_O_3_, supported with 1% FBS) for 3 minutes. Then, the adherent cells were washed twice with PBS and treated with 0.2 ml EDTA-trypsin solution (w, % = 2.5%, Sigma). The cells were suspended with 0.5 ml etchant for 1 minutes, and then collected from removing the supernatant by centrifuging at 1000 rpm, 4 °C. The collected cells were washed twice with PBS and separated at the same centrifuging condition. The whole etching process were carried out at 4 °C to prevent cells from impairing. Cell viability test was carried out after etching within 30 min to evaluate the cytotoxicity of etchants to A549 cell. Details were described in SI. AgNPs suspension was prepared in CM supported with 1% FBS in this study.

**First order removal model.**

$\frac{A}{A_{0}}=(1-\frac{A_{ns}}{A_{0}})\mathrm{e}^{-(\frac{V_{s}}{h}+\mathrm{k}_{\mathrm{dis}})\mathrm{t}}+\frac{A_{ns}}{A_{0}}$ (S1)

Where Vs is apparent sedimentation factor, cm min^-1^; h is the sediment distance, 2 cm in this study (in a 1.25 × 1.25 × 4.5 cm^3^ cuvette); k_dis_ is dissolution factor, min^-1^, considering of poor dissolution of AgNPs in CM, k_dis_ is 0 min^-1^ in this study; t is time, min; A is the absorbance of suspension at t; A_0_ and A_ns_ is the initial absorbance and absorbance of residual suspension which suspension is forward to reach.

**Table S1.** Fitting parameters of first order removal model.

|  | R^2 *^ | V_s_ ^*^  (cm min^-1^) | C_ns_^*^ | V_sh_^**^  (cm min^-1^) |
| --- | --- | --- | --- | --- |
| nAg_control_ | 0.991 | 0.0359 | 16.52 | 0.0079 |
| CA-nAg | 0.973 | 0.0304 | 18.04 | 0.0016 |
| FA-nAg | 0.993 | 0.0326 | 18.48 | 0.0015 |
| TA-nAg | 0.998 | 0.0284 | 18.32 | 0.0009 |

^*^: the sedimentation of 20 μg ml^-1^ AgNPs in a 1.25×1.25×4.5 cm^3^ cuvette; 2 cm for h (half of the liquid height). ^**^: the linear part of M from the total mass of AgNPs; 0.11 cm for h.

**CCK-8 cell viability assay.**

A549 cells were seeded in 96 well-plate for 24 h prior to exposure with AgNPs. Seeding density was 10^4^ cells per well in 200 μl CM with 10% FBS. At the following day, cells were exposed to 100 μl AgNPs suspension for 24 h. The plates were added in 10 μl CCK-8 reagent (Shanghai, Beyotime) per well and then incubated for 20 min. The absorbance was measured by UV-Vis spectrophotometry at 450 nm. To subtract the interference from the absorbance of AgNPs at 450 nm, the absorbance before the addition of CCK-8 reagent was also measured. The concentration of AgNPs were 0 - 100 μg ml^-1^. Six groups were carried out to use in the parallel examination.


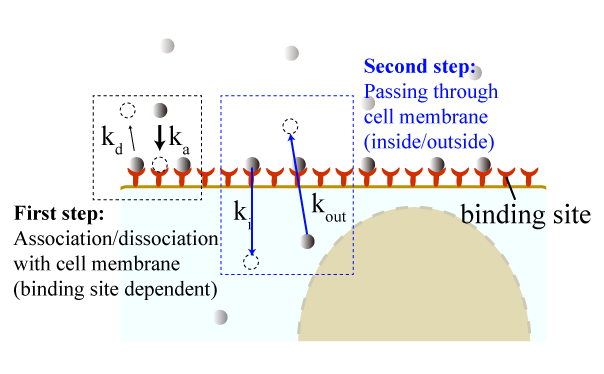


**Fig. S1.** The schematic image of two process of kinetic mechanism of AgNPs internalization into A549 cells.


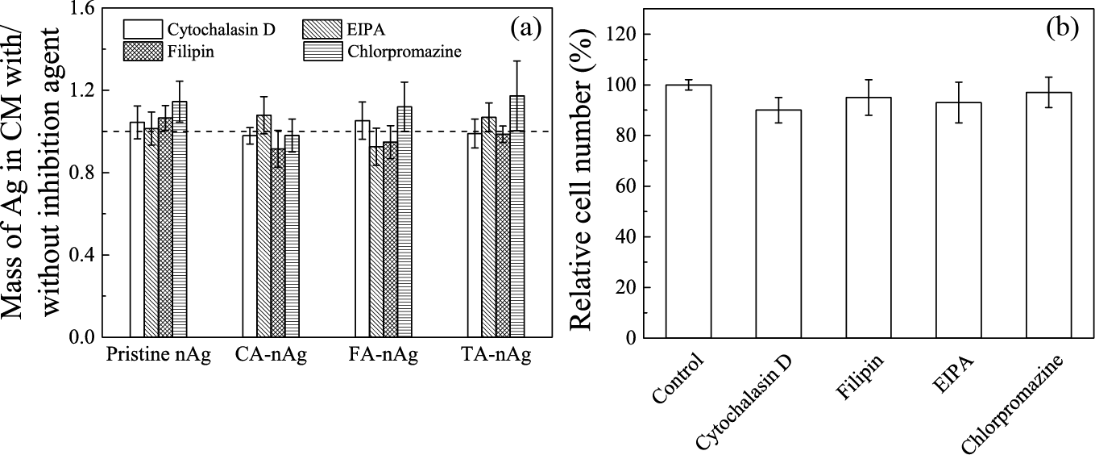


**Fig. S2.** (a) Stability of the AgNPs suspension in culture medium supported with 1% FBS were less impaired by inhibitors incubated over 1 h at 37 °C according to the value of the mass of Ag in CM with inhibitors divide to the mass of Ag in CM without inhibitor. (b) A549 cell number counted by flow cytometry after incubated with inhibitor for 1 hour. Concentration of these inhibitors were 5 μM for cytochalasin D, 5 μM for EIPA, 30 μM for chlorpromazine and 0.5 μg ml^-1^ for filipin.


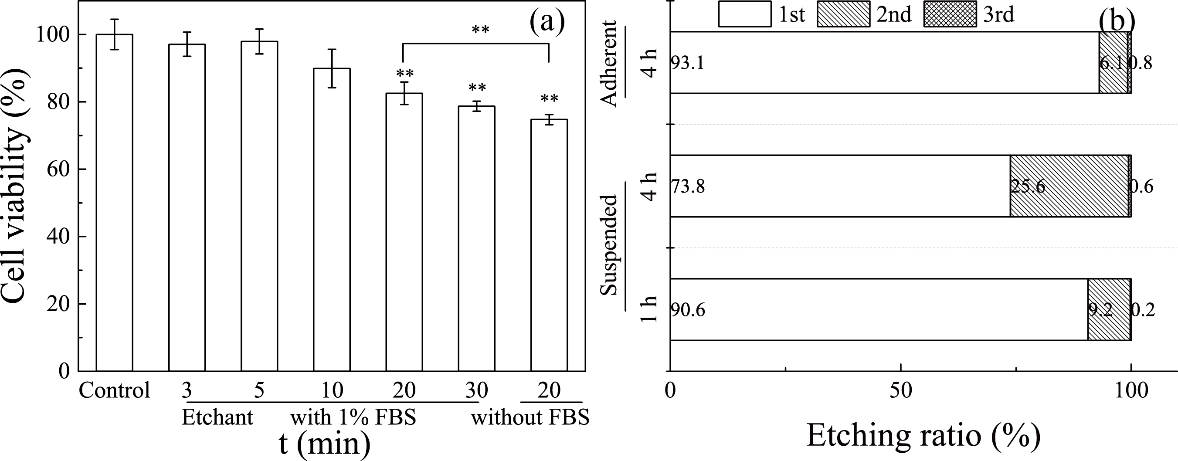


**Fig. S3.** The low cytotoxicity to A549 cell and high efficient to remove AgNPs from cell surface of 10 mM etchants supported with 1% FBS. (a) A549 cell viability (cell number: 20000) in 96 well-plate after contacted with 10 mM etchants at 4 °C with 1% FBS for 3, 5, 10, 20 and 30 min, or without FBS for 30 min (**, p < 0.01). (b) Etching ratios of AgNPs for adherent cells after exposure over 4 h to nAg_control_ washed with 10 mM etchants supported with 1% FBS for 3 times (once for 3 min) comparing to the ratios for cells after exposure over 1 h or 4 h to 10 mg l^-1^ nAg_control_ with the 1st time washing adherent and followed with 2 times washing suspended.


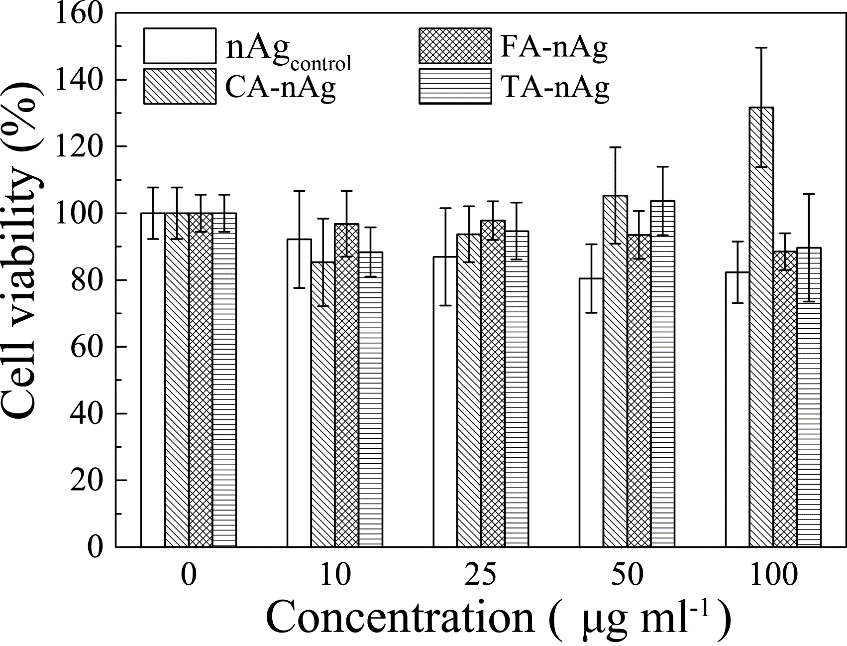


**Fig. S4.** Cell viability to A549 cell after incubated with pristine nAg, CA-nAg, FA-nAg and TA-nAg for 24 h at different concentration.
